# Realist complex intervention science: Applying realist principles across all phases of the Medical Research Council framework for developing and evaluating complex interventions

**DOI:** 10.1177/1356389016652743

**Published:** 2016-06-02

**Authors:** Adam Fletcher, Farah Jamal, Graham Moore, Rhiannon E. Evans, Simon Murphy, Chris Bonell

**Affiliations:** Cardiff University, UK; UCL Institute of Education, UK; Cardiff University, UK; Cardiff University, UK; Cardiff University, UK; London School of Hygiene & Tropical Medicine, UK

**Keywords:** Complex interventions, complex systems, realism, evaluation, pilot trials, randomised controlled trials, public health

## Abstract

The integration of realist evaluation principles within randomised controlled trials (‘realist RCTs’) enables evaluations of complex interventions to answer questions about *what works, for whom and under what circumstances*. This allows evaluators to better develop and refine mid-level programme theories. However, this is only one phase in the process of developing and evaluating complex interventions. We describe and exemplify how social scientists can integrate realist principles across *all phases* of the Medical Research Council framework. Intervention development, modelling, and feasibility and pilot studies need to theorise the contextual conditions necessary for intervention mechanisms to be activated. Where interventions are scaled up and translated into routine practice, realist principles also have much to offer in facilitating knowledge about longer-term sustainability, benefits and harms. Integrating a realist approach across all phases of complex intervention science is vital for considering the feasibility and likely effects of interventions for different localities and population subgroups.

## Introduction

The original UK Medical Research Council (MRC) framework for evaluating complex interventions recommended sequential phases of development, feasibility testing and evaluation, culminating in the estimation of an effect size via a randomised controlled trial (RCT), prior to wider implementation (Campbell et al., 2000). This emphasis on aggregate effectiveness, reflected within many subsequent trials of complex public health interventions, has left trialists open to critiques from ‘realist evaluators’ (for example, Pawson, 2013) that trials oversimplify causality, and are fundamentally unsuited to the evaluation of complex interventions. Effect sizes may tell us that an intervention helped more people than it harmed in the time and place it was delivered, but often tell policymakers and practitioners little regarding how findings might be applied in new settings or to other populations (Cartwright and Hardie, 2012). An emphasis purely on aggregate effectiveness also means that we risk developing, evaluating and recommending interventions for implementation that have small population-level benefits at the expense of widening existing inequalities (Whitehead 2007).

However, the fact that trialists have not historically considered these issues sufficiently does not mean that they cannot. While often presented as opposing factions (Marchal et al., 2013; Pawson and Tilley 1997), experimental social science is highly compatible with the methodological principles and epistemological assumptions of critical realism which underpin realist evaluation (Bonell et al., 2012, 2013a). Critical realism is a philosophy of science founded on the stratification of social reality into the domains of the real, the actual and the observable (Pawson, 2013). Critical realism seeks to support social scientific investigation through a recognition that the object of such investigation must have real, internal mechanisms that can be *actualised* to produce particular social outcomes (Bhaskar, 2008). Evaluation, including through experimental designs, directly supports the scientific observation of such mechanisms, which are activated in certain contexts of the actual, to explain patterns of social causation and problems in the domain of the real (Bonell et al., 2013a).

Realist evaluation focuses on building, testing and refining middle-range theories regarding complex casual mechanisms and how these interact with individuals’ agency and social context to produce outcomes (Hawkins, 2014; Pawson, 2013). The term ‘middle-range theory’ was developed to distinguish grand social theories (e.g. functionalism) from the process of integrating theory and empirical research to explain patterns of social behaviour and outcomes in a particular social setting (Merton, 1968). The development and testing of theories about context–mechanism–outcome (CMO) configurations within realist evaluation is one such example of middle-range theory and research (Pawson, 2013), and this process can build on programme ‘logic models’ that define the components and intended mechanisms of action of specific interventions (Bonell et al., 2012). The most recent MRC guidance on evaluating complex interventions, while maintaining that RCTs should be used to test effectiveness where possible, placed increased emphasis on the use of evaluation to build theory and understand causal mechanisms (Craig et al., 2008a), though the role of context in shaping implementation and causal processes is only briefly mentioned. In particular, aspects of this guidance focussed on intervention development pay no attention to context. Unlike with realist evaluation, there is little emphasis on developing and testing theories.

An emergent field of enquiry within evaluation, which is highly compatible with realist principles and foregrounds the role of context in understanding complex interventions, is complex systems science (Hawe et al., 2009; Westhorp, 2012). Indeed, the MRC guidance has been criticised by some for the use of the term ‘complex’ in the absence of engagement with complexity theories and thinking (Anderson 2008; De Silva et al., 2014). At present, the MRC guidance conceives complexity largely in terms of synergies between intervention components (for example, the added value of combining an educational component with an environmental component). However, Hawe (2015a), who has advocated the use of RCTs in evaluating complex interventions (Hawe et al., 2004; Shiell et al., 2008), argues that we should conceive complexity in terms of how interventions interact with their contexts. A social intervention represents a disruption to complex systems, or attempts to change the dynamics of the systems in which they are delivered, and hence pre-existing contextual factors will shape what is delivered, how it will work, and for whom (Hawe et al., 2009). Using the example of early intervention programmes, Westhorp (2013) has illustrated the compatibility of ‘complexity-consistent theory’ for refining mid-level programme theories about mechanisms of actions and the contexts that activate them.

Thus, there is an inherent compatibility of complex systems science, critical realism and realist evaluation in their mutual commitment to understanding causality within complex environments. Ontologically, these approaches are consistent that causality should be understood as always dependent on the whole context of an intervention, including the complex and emergent systems within which it is embedded (Byrne, 2013). That is to say, causation is a consequence of multiple factors rather than any single specific factor, and will operate in different ways such that the same outcome may be generated by different causal combinations in different contexts. There is also substantial overlap between a complexity approach to evaluation and realist evaluation, due to their explicit concern with social theory and focus on understanding the interplay of agency and structure (Byrne, 2013).

Progress is being made in integrating complex systems science and realist evaluation principles with RCTs through ‘realist RCT’ designs, to allow evaluators to go beyond simply asking ‘does it work’ and towards more nuanced consideration of *what works, for whom and under what circumstances* (Bonell et al., 2012). Large-scale realist RCTs are now being undertaken in the UK (for example, Bonell et al., 2014) and sub-Saharan Africa (for example, Chandler et al., 2013). New MRC guidance on integrating process evaluation within trials of complex interventions also endorses the use of RCTs that integrate qualitative data collection and analysis focussed on the interactions between mechanisms, context and outcomes (Moore et al., 2014, 2015). However, effectiveness trials are only one phase within the process of developing and evaluating public health interventions. In order for realist RCTs to deliver health improvement benefits via developing well-theorised, effective, scalable health improvement interventions, it is vital that other phases of intervention development and refinement are also as clearly focussed on generating knowledge about their mechanisms of action and how these can interact with social context to produce various outcomes.

## Complex intervention science phases

The 2008 update of the MRC guidance for complex intervention development and evaluation provides a four-phase, cyclical framework advising health researchers to answer a range of sequential questions regarding complex intervention theory, feasibility and acceptability, effectiveness and cost-effectiveness, and sustainability (Craig et al., 2008a). The first phase (intervention development) involves the development of an intervention’s theoretical rationale, often depicted in a ‘logic model’ describing inputs that the intervention involves, the processes that these initiate, and the mechanisms via which these are intended to realise positive outcomes. This phase should identify underpinning ‘active ingredients’ and how intervention components are expected to synergistically interact with one another, and with the context of delivery (although less emphasis is given to this), to generate outcomes (both intended and unintended) (Bonell et al., 2015).

The subsequent feasibility and piloting phase includes testing the feasibility and acceptability of the proposed intervention and its evaluation methods. Although the exact distinction between feasibility and pilot studies is contested (Lancaster, 2015), pilot studies may simply be a smaller version of the main trial, aiming to implement the intervention and its trial on a smaller scale (for example, with smaller samples, in fewer sites and/or for shorter follow-up periods), while feasibility studies may focus only on select intervention or trial elements about which there is particular uncertainty. Further refinements may be made to the intervention theory after this phase to optimise the intervention design, logic model and the proposed evaluation design prior to testing effectiveness and cost-effectiveness.

Once a well-theorised intervention has been developed and feasibility questions addressed, RCTs are recommended to examine their effectiveness (and cost-effectiveness) whenever randomisation is practicable (Craig et al., 2008a). Finally, ‘implementation studies’ are also needed to address the scale-up of interventions into routine practice (Craig et al., 2008a). The cumulative effect of these processes should be the generation of a strong theoretical and evidence base for public health intervention which provides greater confidence that outcomes observed during trials can be replicated in real-world settings, and which supports the ongoing cycle of developing and evaluation complex interventions.

This article outlines how realist evaluation principles have much to offer public health intervention science, not only for trials of effectiveness but also across all phases of public health intervention science, from intervention development, feasibility and pilot studies to post-evaluation scale-up studies. For example, as the number and range of feasibility and pilot studies proliferates (Arain et al., 2010; Lancaster, 2015), a realist lens can be applied to such studies to address questions regarding not only what is feasible and acceptable in general, but also for whom and under what circumstances, and place much more emphasis on exploring potential mechanisms of action (i.e. the intermediate processes triggered by the introduction of an intervention, which give rise to intended, and unintended, consequences) and how these may vary by context prior to large-scale realist RCTs. This is vital in ensuring that we are clear via what mechanisms and in what contexts interventions are expected to work, and for whom, and focus later phases of evaluation on interventions that have potential to be deliverable in the most salient settings, effective for key populations, and are scalable. Once realist RCTs of complex interventions have demonstrated their effectiveness, subsequent realist evaluations of their scale-up should enable us to further refine our understanding of how these interventions play out in an even greater diversity of contexts. This will better inform attempts to adapt implementation to local conditions while ensuring consistency with the core theoretical principles of the intervention.

Some of the authors of the revised MRC guidance have subsequently argued that approaches such as complex systems science and realist evaluation may become routine within public health evaluation methods once sufficient empirical examples are available to guide practice (Craig and Petticrew, 2013). This article draws on new case examples of realist studies across the different phases within the latest MRC guidance (Craig et al., 2008b) to provide guidance on the theoretical and methodological process of integrating a realist approach throughout this cycle of intervention development and evaluation. Each phase of intervention science is considered in turn: from intervention development and feasibility and pilot studies, to subsequent evaluations of intervention effectiveness, and implementation studies of scaled-up interventions. We conclude by discussing what structures and partnerships are also required to facilitate realist intervention science, such as the development of specialist social science trials infrastructure to embed these principles within public health evaluation science, and further investment in transdisciplinary research networks to support the quantity, quality and relevance of realist intervention science (Glasgow et al., 2003; Stokols, 2006).

## Intervention development and modelling

Within the revised MRC guidance, there is relatively little attention paid to the developmental phase of the complex intervention cycle (Craig et al., 2008a,b). Other frameworks and toolkits have been developed to specifically support intervention development but these tend to ignore the complexity of multi-component, and particularly multi-level, approaches to health improvement and also the importance of considering context (Hawe, 2015b). For example, the literature providing guidance on the development of intervention logic models is still informed by simple, linear behaviour–determinant–intervention (BDI) toolkits (e.g. Kirby, 2004) and ignores how implementation and causal pathways may vary by context (for example, ‘intervention mapping’ as proposed by Bartholomew et al., 2011).

More recently, theoretically orientated tools have been developed, such as the ‘Behaviour Change Wheel’ (Michie et al., 2011) and the ‘Theory of Change tool’ (De Silva et al., 2014) with the aim of improving public health intervention development. However, these focus on helping researchers and practitioners categorise and label intervention inputs and activities more systematically, which overprivileges parsimony and oversimplifies complex social realities. These tools also do not engage with a realist approach focussed on theorising mechanisms nor how these vary by context. These approaches also tend to suggest an idealised and highly linear sequence in which, for example, all objectives and pathways are pre-specified prior to designing components and planning implementation, which, first, ignores the potential of retrospective theoretical modelling of existing interventions and, second, overlooks the likelihood that all mid-level programme theories will need to be iteratively tested and refined in the light of subsequent pilot and evaluation findings.

Addressing these existing gaps in the literature and via engagement with a realist lens, we recommend further development and use of the following three methods to support intervention development and modelling: mixed-methods evidence synthesis; formative mixed-method, multi-case-study research; and, pragmatic formative process evaluation. These methods would support the development of more three-dimensional (3-D) logic models, which focus not only on complex the pathways from (1) inputs to (2) outcomes but also the (3) contextual dimensions that activate or mitigate causal processes. Intervention logic models (referred to as implementation models by Weiss, 1995) have typically focussed on defining the components and mechanisms of specific interventions within a very particular setting and paid relatively little attention to how mechanisms interact with context and produce potentially contradictory processes and outcomes in different localities and for various populations subgroups (Bonell et al., 2012; Moore et al., 2015). The inclusion of a contextual dimension within the logic models at the intervention development stage would in turn support the subsequent phases of realist evaluation, which are outlined later in this article.

### Mixed-method evidence synthesis

The process of designing more theoretically driven interventions and specifying potential CMO configurations has been hindered by the dominant paradigm within evidence syntheses: systematic reviews still typically focus on synthesising only quantitative studies answering questions about ‘what works’ at the expense of understanding how, in what context and for whom (Pawson, 2013; Petticrew, 2015). These evidence reviews therefore still typically only focus on accrediting public health policies and interventions as ‘effective’ (or otherwise). Methods such as meta-analysis traditionally aggregate across studies to derive overall effect sizes, rather than exploring how and why trials of similar interventions produce different outcomes in different contexts. The dominance of such reductionist methods is associated with the rise of intervention-comparison websites (similar to price-comparison websites), such as the *Blueprints Youth Programmes* resource developed in the USA (http://www.blueprintsprograms.com/) and the UK *Investing in Children* database (http://investinginchildren.eu/), which accredit lists of ‘effective’ interventions without consideration of which contexts such interventions might be suitable.

Mixed-methods reviews have similarities with mixed-methods primary research, thus there are many ways in which the products of different syntheses methods can be combined to overcome the limitations with traditional systematic review methods. ‘Realist reviews’ have been suggested as an alternative (or adjunct) to address the lack of focus on CMO configurations in current evidence syntheses (Pawson et al., 2005). However, although realist review guidelines include a stronger focus on examining context as well as outcomes (Wong et al., 2013) and can provide a conceptual platform prior to complex intervention development (Pearson et al., 2015a), they are more open ended and often not do involve an a priori protocol. Such protocols are necessary to minimise bias and retain practical focus, and this has limited the potential of realist reviews to support the development of practical, theoretically driven, population-level health improvement interventions. As with realist trials (Bonell et al., 2012; Jamal et al., 2015), it is possible for systematic reviews to be guided by a priori protocols while being mixed method and thus more attentive to mechanism and context. To do this, reviews can continue to synthesise evidence of overall effects from RCTs and quasi-experimental studies (including via meta-analysis where appropriate) while also undertaking other syntheses to better understand how interventions work and how this might vary with context. There are two main ways of doing this.

First, reviews can synthesise information on theories of change and evidence on intervention processes to develop hypotheses about the mechanisms via which interventions are intended to work, as well as how implementation and effectiveness might be affected by the characteristics of different populations and places. For example, two recent mixed-methods reviews – one examining how the school environment and school–environment interventions influence health, and one examining the effects of community-based positive youth development (PYD) interventions – have synthesised intervention theories and the findings from process evaluation reports as well as estimates of intervention effects to hypothesise how school environment and PYD interventions can improve health, for whom and in what contexts (Bonell et al., 2013b, 2016). A realist systematic review and synthesis of studies examining the process of implementing health programmes in schools also highlights the benefits of reviewing process data systematically to develop programme theories and support intervention design (Pearson et al., 2015b). This method allowed the authors to identify transferable mechanisms that support implementation when preparing for, and introducing, new programmes in a school.

Second, reviews can use meta-regression or qualitative comparative analysis (QCA) (Ragin et al., 2006; Thomas et al., 2014) to examine how intervention effects vary according to the characteristics of settings or populations, or examine intervention effects on potential mediators and whether these might account for effects on primary outcomes. With both of the school environment and PYD reviews cited above, the intention was to use the hypotheses derived from syntheses of theories of change and process evidence to inform selection of which moderator and mediator variables to examine in syntheses of outcome evaluations. In neither case was this possible because the included outcome evaluations did not report potential moderators or mediators consistently enough to allow syntheses to examine these. However, other reviews, while not using preliminary syntheses of theoretical literature and process evidence to inform hypotheses, have been able to test what contextual factors appear to moderate intervention effectiveness. For example, a review and meta-analysis of criminal justice interventions by Lipsey (2009) examined how the site of delivery moderated effectiveness. QCA has also been tested and allowed reviewers to go beyond basic, narrative synthesis of integrated process evaluations and identify key intervention characteristics and how effects may occur (for example, Thomas et al., 2014). Such methods of evidence synthesis will be facilitated as more studies adopt a realist lens, as outlined in the discussion.

### Formative case studies

As well as mixed-methods systematic reviews to identify the relevant theoretical and evidence base, before new interventions are piloted it is often useful to undertake formative, mixed-method case-study research to understand their socio-ecological context, explore potential intervention delivery and hypothesise mechanisms of action. Such formative case studies can employ purposive sampling to provide contextual diversity, informed by initial theories, and generate insights regarding how these contexts might interact with intervention mechanisms to influence outcomes for different groups.

One example of this design is a current formative study to develop and model a new intervention to be delivered in further education (FE) colleges to promote safe sex and relationships among 16–19-year-olds. Six FE colleges in England and Wales were purposively sampled according to type and size of institution. A phased approach to data collection and analysis supports the consideration of CMO. First, focus groups and interviews have been used to explore the views of students, teachers, managers and sexual health service providers on how interventions deliverable within FE colleges might work to improve relationships and sexual health. Second, informed by these data, a larger cross-section of students and staff were surveyed to develop theories about how these mechanisms might interact with context to play out differently in different settings and/or with different groups of students (for example by gender, sexuality, socioeconomic status (SES) and/or baseline sexual risk). Finally, findings from these elements will be brought together to refine a 3-D intervention logic model which incorporates consideration of CMO configurations.

The design and development of a new film-based intervention targeting teenage men to prevent unintended pregnancy has also involved formative, mixed-methods research in a range of settings (Aventin et al., 2015). To develop a theoretical understanding of the phenomenon of unintended teenage pregnancy in relation to young men – who are not typically targeted by teenage pregnancy prevention interventions – a mix of methods was necessary, including consultations with schools, focus groups and a survey to assess the views of a wider cross-section of young men aged 14–17 about potential intervention components. A strength of this study is that it went beyond the basic MRC guidance on developing complex interventions by also explicitly addressing contextual complexities through engaging a range of the target group (young men) across a range of settings (schools) (Aventin et al., 2015).

### Pragmatic process evaluations

The development of new interventions and modelling of theories of change can also be enhanced by pragmatic process evaluations of interventions already in routine practice (Evans et al., 2015a). Although such evaluations remain somewhat rare, these designs allow us to move beyond the theorisation of how a postulated theory of change may play out in real-world settings as intervention mechanisms are already interacting with contextual characteristics across a range of settings: the ‘C’ element of CMO configuration is already privileged within pragmatic, formative evaluations (Evans et al., 2015a).

These evaluations allow for the examination of mechanisms not only of intended benefits but also unanticipated consequences, including unintended harms. For example, a pragmatic formative process evaluation of a school-based social and emotional learning intervention identified a number of iatrogenic effects as a consequence of the stigmatising referral processes and negatively labelling young people (Evans et al., 2014). Through using a mixture of direct observations and interviews with multiple stakeholders to capture their different perspectives, these studies also provide insights into the organisational-level barriers and facilitators of implementation (Evans et al., 2015b). Whereas the MRC progression framework has tended to address implementation and translational issues at the point of scale-up following a trial, pragmatic process evaluation of existing interventions allow this to be theorised and empirically explored from the start, which will help to ensure intervention development studies have external, and socio-ecological, validity and supports more sustainable implementation procedures.

Our suggestion is not that resources should be used to retrospectively theorise all existing interventions on an exhaustive basis. However, once existing interventions are deemed to warrant outcome and process evaluation they should be first subjected to pragmatic formative process evaluation to help develop the intervention logic model, model realist CMO hypotheses and, if necessary, refine delivery methods prior to larger-scale evaluation and scale-up. Without a clear theory of change, subsequent evaluations employing a realist perspective will be of more limited value. One example of where an existing but under-theorised intervention was subjected to pragmatic process evaluation was the Welsh National Exercise Referral Scheme (NERS) (Murphy et al., 2012). Theoretically informed analyses of the trial data were able to examine variations in health benefits across different groups, and contextual interactions, which are described below (‘Realist RCTs’) as an illustration of the benefits of integrating realist principles across multiple evaluation phases.

## Realist feasibility and pilot studies

Feasibility and pilot studies should also apply a realist approach to explore implementation and potential mechanisms of action in a range of contexts prior to larger effectiveness trials. Following the development of MRC guidance on complex interventions (Campbell et al., 2000; Craig et al., 2008a), the volume of feasibility and pilot studies, particularly pilot RCTs, has increased markedly (Arain et al., 2010; Lancaster, 2015). Such preliminary studies of theoretically informed interventions provide an opportunity to examine barriers and facilitators to implementation in a range of settings, to explore the views of those involved, and to refine and optimise the intervention design, logic model and trial methods prior to realist RCTs. However, to date, pilot RCTs have often only answered relatively crude, binary questions about whether a specific complex intervention is feasible and acceptable, or not.

The dominance of such binary assessments is now reflected in the widespread use of binary ‘progression criteria’, including by funders, to determine whether a subsequent, larger evaluation is warranted (e.g. Newbury-Birch et al., 2014). Feasibility and pilot studies should instead assess what is feasible and acceptable for whom and under what circumstances, aiming to refine hypotheses about potential mechanisms of action and how these might vary by context, and pilot the methods and measures that can capture these. Several realist strategies have been used and should be developed and used more widely at this stage in the cycle of intervention development and evaluation to refine intervention theories and support subsequent, large-scale realist evaluation studies testing programme theories.

First, purposive sampling criteria should be used in pilot RCTs to ensure there is sufficient diversity in aspects of context that have been pre-hypothesised to affect feasibility, acceptability and causal mechanisms. It is essential to assess these in a range of contexts, but this rarely happens in practice. One example is a pilot cluster RCT of whole-school restorative approach to prevent bullying and aggression in secondary schools (Fletcher et al., 2015). This study used a purposive sampling matrix to recruit a theoretically informed diversity of schools that varied according to the SES of their students (high/low free school meal eligibility) and inspectorate rating of school ‘effectiveness’. This study also purposively sampled a range of more or less experienced intervention delivery staff. In the case of pilot trials in which individuals, rather than clusters, are the unit of allocation, there is still a need to encompass relevant diversity in intervention sites and individuals. Exploration of contextual variation in feasibility and acceptability at this stage also allows researchers to identify ways in which the intervention delivery might be adapted to different contexts if necessary (while maintaining consistency with underlying theory).

Second, like subsequent realist RCTs (as outlined in Bonell et al., 2012), feasibility and pilot trials provide the opportunity to collect and analyse rich qualitative data to support the refinement of hypotheses about causal pathways to test in subsequent effectiveness trials. Feasibility and pilot studies also do not aim to estimate intervention effects, so research teams can collect much more data, especially qualitative data, from intervention or control groups without concerns about this biasing outcome measurement, for example via Hawthorne effects. A specific progression criterion from pilot to large-scale trials should focus on the refinement of hypotheses in this way.

Third, where appropriate, multi-arm pilot RCTs can be employed to help assess the feasibility, acceptability and potential mechanisms of multiple different interventions, or to pilot multiple intervention components separately. A four-arm cluster randomised pilot trial in 12 secondary schools in south Wales is being used to assess the feasibility, acceptability and potential impacts of different peer-led drug-prevention intervention methods (White et al., 2014). As well as piloting the use of a control group, there are three different ‘intervention arms’: ‘ASSIST’, an existing peer-led smoking-prevention intervention targeting year 8 students (aged 12–13); ‘ASSIST+Frank’, which combines ASSIST with a new informal peer-led drug-prevention adjunct targeting year 9 students (aged 13–14); and ‘Frank friends’, which is a new stand-alone, informal drug-prevention intervention delivered in year 9. The embedded process evaluation will explore the views of students and school staff regarding the two different pilot methods of delivering peer-led drugs education (‘ASSIST+Frank’; ‘Frank friends’), and assess implementation fidelity by arm. Depending on the results of piloting, these multi-arm designs may or may not be taken forward as multi-arm, realist RCTs, or it may be decided to merge or remove arms.

## Realist RCTs

The term ‘realist RCT’ has been used to describe large-scale mixed-method trials that combine the advantages of the minimisation of bias in estimating intervention effects via randomisation to a control group, with the ability to theorise the mechanisms underlying these effects as well as how effects differ by social group and place (Bonell et al., 2012, 2013a). This combination means that realist trials maximise internal validity in estimating effects within the trial (and how these are moderated by contextual factors) as well as maximising external validity by developing evidence-based theories about the factors which will promote or limit the effectiveness of the intervention in other settings and with other populations. New MRC process evaluation guidance supports the combination of RCT methods with detailed process evaluation to understand mechanisms and context (Moore et al., 2014, 2015), although there are few examples of such studies to date.

One such example is the Welsh NERS policy trial that built on a pragmatic, formative mixed-method process evaluation to develop the intervention logic model (Moore et al., 2012). In the trial of the NERS, quantitative and qualitative data were then used to test and refine the programme theory. For example, a key hypothesised mechanism for improving physical activity was increased autonomous motivation. Several components targeting this mechanism were not well delivered (Moore et al., 2013). Nevertheless, mediation analyses showed that change in physical activity appeared to be explained by change in autonomous motivation (Littlecott et al., 2014). It appears from qualitative data that this mechanism was triggered largely by emergent social aspects of the scheme rather than by motivational counselling techniques (Moore et al., 2013). Moderation analyses were also able to examine how effects varied according to subgroups, which found that the programme did not increase physical activity for those patients referred for mental health reasons but did for those referred on the basis of coronary heart disease risk (Murphy et al., 2012). Aforementioned qualitative process data enabled researchers to understand the social processes through which patterning in responses to the intervention emerged.

A realist RCT of a whole-school restorative approach to preventing bullying, which followed the earlier realist pilot RCT described above, is developing and using a three-stage theoretical and methodological process of building and testing mid-level theories (Jamal et al., 2015). First, informed by the findings of the prior pilot study and sociological theory, researchers elaborated the theory of change and specific a priori hypotheses about CMO configurations. Second, emerging findings from the integral process evaluation within the RCT are being used to refine, and add to, these a priori hypotheses before the collection of quantitative, follow-up data. Third, hypotheses are tested using a combination of process and outcome data with quantitative analyses of effect mediation (examining mechanisms) and moderation (examining contextual contingencies). The main output of the RCT is to assess whether the intervention is effective or not, but importantly to also refine and further develop an empirically informed theory of change. This process also supports evaluators to identity both intended and unintended consequences of complex interventions, including through iteratively developing and testing ‘dark logic models’ (Bonell et al., 2015).

A realist approach to trial design also helps draw greater attention to how aspects of usual care (i.e. the control group condition) may foster mechanisms similar to the intervention in some contexts, which is rarely considered by trialists at present. For example, a meta-analysis of studies examining adherence to HIV care concluded that between-study variation in intervention effectiveness could be explained as much by differences in behaviour change elements in the usual care arms of the included studies as by variation in interventions (De Bruin et al., 2010). More fully theorising comparison-group contexts, as well as building and testing programme theories, is particularly important for fostering appropriate cross-national and cross-cultural replication of programmes. For example, the Family Nurse Partnership programme, an intensive model of prenatal and early childhood home visiting for vulnerable first-time mothers and their children found to be effective in the USA (Olds, 2016), has been replicated and trialled at scale in England with no benefits observed (Robling et al., 2016). Post-hoc theorisation of the programme has focussed on variations in pre-existing community contexts (i.e. control group care), as well as the programme itself, and how the null effects observed in a UK context could be attributed to all mothers having free access to a range of supportive health and social services (Olds, 2016; Robling et al., 2016). To put this another way, the powerful effects observed in the USA appear to be fired through the programme mechanisms interacting with the more ‘Darwinian’ nature of usual care in that context, with little state support for poor, young mothers for whom the greatest effects were observed.

## Scale-up evaluations

Realist approaches can also be applied where interventions are scaled up after successful trials. Evaluations of scale-ups can examine long-term benefits and harms and how these vary by context. These studies can occur over a wider range of settings, populations and time periods and so have particular strengths in understanding how context shapes outcomes.

One example of this is the evaluation of the scale-up of the Intervention with Microfinance for AIDS and Gender Equity (IMAGE), which did not explicitly use a realist approach but nonetheless embodied some of its key principles. The IMAGE intervention combined group-based lending with gender and HIV education, and facilitated community mobilisation campaigns, targeting women living in poverty in rural South Africa. Following a cluster RCT trial that suggested that this was effective in reducing rates of intimate partner violence (Pronyk et al., 2006), this intervention was scaled up to other rural sites within South Africa. The follow-on scale-up evaluation did not aim to examine effectiveness but built on the process evaluation embedded within the cluster RCT to examine longer-term implementation processes and potential mechanisms in contrasting sites (Hargreaves et al., 2010). This study suggested that community mobilisation components were often not sustainable, particularly in those contexts where women were targeted on the basis of poverty and were socially marginal within the villages in which they lived. Community mobilisation was intended to reduce sexual risk behaviours among women’s household members and villagers via a mechanism involving increased critical consciousness of the social determinants of risk. The evaluation’s finding that this mechanism may not have been functioning in some contexts provided insights into why IMAGE may only have been effective for the women themselves and enabled refinement of the theory of change.

There are few, if any, other examples of such MRC ‘implementation’ studies using realist approaches, although there are examples of ‘natural experiments’ of large-scale interventions using realist approaches (e.g. Humphreys and Eisner, 2014). However, if realist principles come to be applied throughout earlier phases of intervention development and evaluation, there will be greater scope for them to inform wider scale-up and ongoing monitoring.

## Discussion

Public health evaluators have typically under-theorised and under-researched how interventions are intended to engage with their social contexts to enact change (Hawe, 2015a; Macintyre and Petticrew, 2000; Moore et al., 2015). If evaluators continue to under-theorise interventions, focus on binary notions of feasibility and acceptability to the neglect of how this is affected by context, and conceptualise complexity only in terms of the number and interaction of intervention components, it is unlikely that their work will amount to a body of intervention theory and scientific knowledge that is useful to policymakers and practitioners who need to know what interventions should be delivered where, how and to whom. A history of what has worked in one time and place cannot be naively treated as a guarantee of future success elsewhere.

While realist RCTs are becoming more common, large-scale outcome evaluations are only one phase in the process of identifying effective, sustainable interventions to improve health. It is also much more difficult to undertake realist RCTs and scale-up studies without earlier phases of development and piloting that develop and refine programme theories and CMO hypotheses. To facilitate a step-change in the quantity and quality of realist RCTs, the development of complex interventions and their theories of change, and preliminary feasibility and pilot studies, should also now adopt a realist focus on context and mechanisms of actions. Purposive sampling is particularly important to ensure a range of contexts are studied at an early stage and the role of context is therefore theorised alongside the intervention logic model. It is then possible to test hypothesised mechanisms of actions (mediation analyses) and examine how outcomes vary by subgroup and place (moderation analyses) within large-scale realist RCTs, as well refining and building new hypotheses within these trials via qualitative data. In some cases, it may also be possible to test moderated mediation (i.e. whether there is an effect mediated by certain mechanisms only under specific contexts), which remains rare in RCTs.

Adopting such a realist approach across all phases of intervention science is vital for considering the likely effects of interventions on different social groups and addressing inequalities in health and other outcomes. For example, at the stage of developing interventions and modelling their mechanisms, it is important to theorise the processes and outcomes for different sub-populations. If more complex logic models are not developed to embrace system-focussed theory it is unlikely that new interventions will respond effectively to the most entrenched social problems and reduce inequalities (Hawe, 2015b). Feasibility and pilot studies should also include a strong focus on implementation and its acceptability among the most deprived communities to ensure that interventions are feasible and sustainable in such contexts. Realist trials that include moderation analysis to assess variation by SES and place can also help to ensure that we do not develop, evaluate and implement interventions that will exacerbate health inequalities in the future.

The major barrier to formally testing CMO configurations within individual studies are the small sample sizes that trials often use, powered to examine effects on primary outcomes but not necessarily sufficiently powered to detect differences in all secondary or intermediate (process) outcomes. Trials are rarely designed with secondary analyses according to mediators or population subgroup in mind (Petticrew et al., 2012), and clinical trials units often reject such secondary data analyses for fear of false positive results and accusations of ‘data dredging’ (Davey Smith and Ebrahim, 2002). We would argue strongly that secondary analyses such as those proposed above are important for a full understanding of how interventions work and for whom, although all analyses should be guided by a priori hypotheses set out in protocols. Even where single studies lack the power for such analyses, reporting their results is useful because it then allows these to be used within systematic reviews and meta-analyses. To facilitate this, studies on related interventions and outcomes should as far as possible use common, validated measures.

If RCTs that adopt realist principles become increasingly common, there is also a need for infrastructure investment to develop the procedures for conducting realist analyses (while avoiding data dredging), facilitate and coordinate new studies, and to develop guidance for developing and reporting robust intervention theory of change. First, there is potential for social science trials teams with expertise in realist methodologies to operate within existing clinical trials units to combine expertise in trial statistics and realist approaches for social interventions.

Second, further investment in transdisciplinary research networks – which involve researchers from multiple disciplines, policymakers, practitioners and the public – is required to increase the quantity, quality and relevance of realist intervention science. This transdisciplinary approach limits the problems created by the separation of the research community from policy and practice, including the concentration of academics on efficacy trials that have little impact on practice (Glasgow et al., 2003; Stokols, 2006). Informed by primary care research networks, which facilitated research capacity (Griffiths, et al., 2000) and fostered a culture of practitioner-led enquiry (Thomas and White, 2001), the Public Health Improvement Research Network (PHIRN) in Wales is one example of a transdisciplinary network that has addressed the limited research capacity, skills and experience of policymakers and practitioners in pragmatic realist complex intervention science. Between 2006 and 2014 PHIRN supported 122 multidisciplinary and multi-sectoral research development groups and secured 72 externally funded research projects focussed on developing and evaluating complex health improvement interventions, including several of the studies cited above (Evans et al., 2014, 2015b; Moore et al., 2013; Murphy et al., 2012; White et al., 2014). As well as increasing the numbers of trials, such co-production can also facilitate mixed-methods reviews of complex interventions (Pearson et al., 2015b; Petticrew et al., 2013) and pragmatic formative studies (Aventin et al., 2015; Evans et al., 2015b). However, there is concern that new UK anti-lobbying regulations may limit, rather than facilitate, knowledge exchange between policymakers and researchers in the future (Smith et al., 2016).

Third, protocol and reporting guidelines should aim to facilitate a step-change towards the realist complex intervention science methods recommended above. For example, trial protocols should include pre-specified moderator and mediator analysis but also allow for iteration in order to refine hypotheses during a trial in light of emerging qualitative data (Bonell et al., 2014; Jamal et al., 2015). Guidance on reporting trials should also include pre-hypothesised mechanism and moderators, for example, within the extension of the CONSORT statement for social and psychological interventions (Mayo-Wilson et al., 2013). Consistent reporting would further support replication studies and systematic reviewers aiming to integrate theory and process data alongside outcome data. Systematic reviewers synthesising social interventions may also value extensions of quality assessment tools (e.g. AMSTAR) that consider key aspects of realist trials principles (e.g. elaborated theory of change, quantitative syntheses of moderator and mediator analyses, and/or QCA). The Cochrane Collaboration’s tool for assessing risk and bias should also be reviewed (Higgins et al., 2011); it currently focuses on internal validity with little consideration for how to reliably synthesis evidence about intervention theory and generalisability beyond the trial setting.

These investments in a realist complex invention science infrastructure and new reporting guidelines would support the cost-effective use of evaluation research funding, and the development of policy-relevant evidence to improve health. Significantly, such an approach offers a way to fully theorise and promote progression through the phases in the MRC framework for the development and evaluation of complex interventions. In turn, greater use of realist RCTs and scale-up studies will, in the long term, support new evidence syntheses that answer a wider range of questions about what works, for whom and under what circumstances, and what carries on working once scaled up and sustained. Those developing interventions or describing their intended mechanisms of action can then draw on such reviews to think more clearly about intended mechanisms and how these interact with context to enable outcomes to manifest.
